# Prediction of Three-Dimensional Arm Trajectories Based on ECoG Signals Recorded from Human Sensorimotor Cortex

**DOI:** 10.1371/journal.pone.0072085

**Published:** 2013-08-21

**Authors:** Yasuhiko Nakanishi, Takufumi Yanagisawa, Duk Shin, Ryohei Fukuma, Chao Chen, Hiroyuki Kambara, Natsue Yoshimura, Masayuki Hirata, Toshiki Yoshimine, Yasuharu Koike

**Affiliations:** 1 Precision and Intelligence Laboratory, Tokyo Institute of Technology, Yokohama, Japan; 2 Department of Neurosurgery, Osaka University Medical School, Osaka, Japan; 3 ATR Computational Neuroscience Laboratories, Kyoto, Japan; 4 Division of Functional Diagnostic Science, Osaka University Graduate School of Medicine, Osaka, Japan; University of Montreal, Canada

## Abstract

Brain-machine interface techniques have been applied in a number of studies to control neuromotor prostheses and for neurorehabilitation in the hopes of providing a means to restore lost motor function. Electrocorticography (ECoG) has seen recent use in this regard because it offers a higher spatiotemporal resolution than non-invasive EEG and is less invasive than intracortical microelectrodes. Although several studies have already succeeded in the inference of computer cursor trajectories and finger flexions using human ECoG signals, precise three-dimensional (3D) trajectory reconstruction for a human limb from ECoG has not yet been achieved. In this study, we predicted 3D arm trajectories in time series from ECoG signals in humans using a novel preprocessing method and a sparse linear regression. Average Pearson’s correlation coefficients and normalized root-mean-square errors between predicted and actual trajectories were 0.44∼0.73 and 0.18∼0.42, respectively, confirming the feasibility of predicting 3D arm trajectories from ECoG. We foresee this method contributing to future advancements in neuroprosthesis and neurorehabilitation technology.

## Introduction

A number of prominent brain-machine interface studies have arisen, in which electroencephalography (EEG), magnetoencephalography (MEG), electrocorticography (ECoG), and intracortical microelectrode have been applied to neuroprosthesis control, neurorehabilitation and novel communication tools for paralyzed or “locked–in” patients suffering from neuromuscular disorders. Since EEG and MEG are non-invasive and have high temporal resolution, they have been used in various paradigms, such as online control of a computer cursor [1]–[2], direction inference of hand movements [3]–[5], operation of a spelling device [6], and neurofeedback for rehabilitation [7]–[13]. Although a large proportion of these non-invasive methods succeeded in classification of movement direction or intention, prediction of time-varying trajectories is likely difficult due to insufficient spatial resolution and low signal-to-noise ratio in such methods.

Signal recording with intracortical microelectrodes is a powerful tool to realize precise trajectory prediction or accurate device control. Using motor cortical signals in animals, studies have shown successful prediction of hand trajectories [14]–[16] and grasp types and velocity [17], control of a computer cursor [18] or a robot arm [19]–[22], and controlled stimulation to a paralyzed arm [23]. These techniques have also been applied in humans to control a cursor [24] and a virtual keyboard and virtual hand [25]. However, though intracortical electrodes can provide rich information for BMI control, they face limitations such as signal degradation due to glial scarring [26]and potential displacement from the recording site [27].

Conversely, ECoG is less invasive than microelectrodes and can offer higher spatial resolutions than EEG and MEG. Researchers have been applying ECoG in humans for several years now and in numerous applications. The classification of hand movement directions or grasp types [28]–[33], one-, two-, or three-dimensional cursor control [27], [34]–[40], and prediction of finger flexion [41] are just some examples of ECoG applications in human patients. Studies concerning the prediction of three-dimensional (3D) trajectory or muscle activities from primate ECoG have shown outstanding results [42]–[45]. Investigations on the prediction of 3D arm trajectory using ECoG in humans, however, are lacking, despite the potential to provide significant improvement in neuroprosthesis and neurorehabilitation technology. The inadequate quality of ECoG signals recorded from patients is one potential obstacle in predicting 3D trajectories. Specifically, (1) paralyzed or elderly patients may find it difficult to perform a long series of repeating trials and stably replicate the same motion for each trial, (2) ECoG signals in patients can include pathological activity, depending on the condition, and (3) the electrode sites on the cortex and the recording lengths can differ, depending on the treatment.

The aim of this study was to predict 3D arm trajectories from ECoG time series in human patients as a basis for a neuroprosthesis. Patients diagnosed with thalamic hemorrhage, ruptured spinal dural arteriovenous fistula (dAVF) and intractable epilepsy executed rotating tasks with three objects on a table. We simultaneously recorded arm trajectories and ECoG signals from 15∼60 electrodes on the sensorimotor cortex. Using a novel method, we predicted four joint angles for the shoulder and elbow joints and six coordinates for the elbow and wrist joints in patients with different pathology.

## Materials and Methods

### Ethics Statement

The study was approved by the ethics committee of Osaka University Hospital (Approval No.08061) and conducted in accordance with the Declaration of Helsinki. ECoG electrodes were embedded not for our experiments but for patients’ medical treatments. Written informed consent was obtained before initiating any research procedures. All patients or their guardians gave written informed consent for the use of their data in the academic study.

### Participants

Three patients (males; 14–64 years) participated in our study (Table 1). Patients 1 and 2 had spastic paresis and weakness in the left arm due to stroke. Their sensorimotor cortices were undamaged, though moderate motor dysfunction was observed. The youngest participant, patient 3, was diagnosed with intractable epilepsy but did not show motor dysfunction. As part of their treatments, all participants were implanted with subdural electrode arrays (Unique Medical Co., Tokyo, Japan) covering the sensorimotor cortex, including the central sulcus. The arrays remained implanted in the intracranium for two weeks to determine the optimum site for effective pain reduction (patients 1 and 2) or epileptic foci localization (patient 3).

**Table 1:** Clinical profiles in patients who participated in this study.

No.	Age	Sex	Diagnosis (Left/Right)	Duration of disease	Paresis (MMT)	Sensation
1	64 yr.	Male	Thalamic hemorrhage (R)	7 yr.	Spastic (4)	Hypoesthesia
2	65 yr.	Male	Ruptured spinal dural arteriovenous fistula	8 yr.	Spastic (4)	Hypoesthesia
3	14 yr.	Male	Intractable epilepsy (R)	7 yr.	None	Normal

### Behavioral Tasks

**Figure 1:**
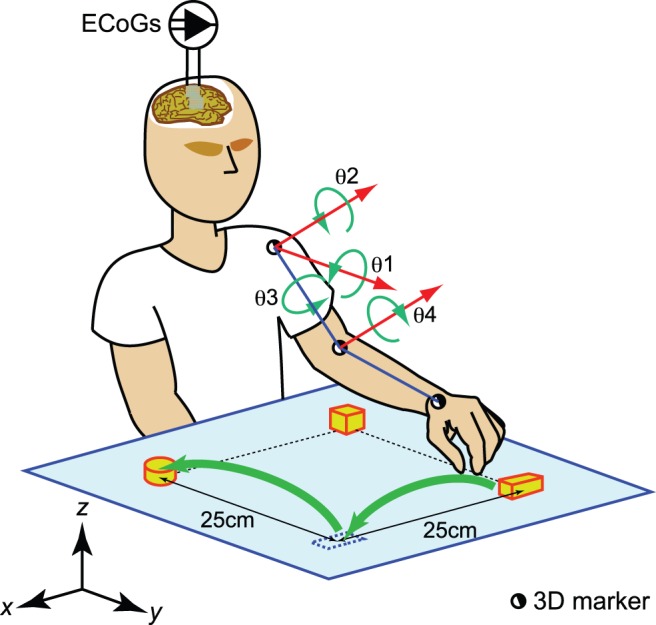
Behavioral tasks. Patient 1 repositioned three blocks one by one and clockwise (green arrows ) at the corners of a 25 cm × 25 cm square. ECoG signals were obtained with planar-surface platinum grid electrodes placed on the right sensorimotor cortex. Half-closed circles on the left shoulder, elbow, and wrist joints represent three-dimensional markers for the motion capture system. The angles q1, q2, q3, and q4 are defined as an abduction/adduction angle, a flexion/extension angle, an external/internal rotation at the left shoulder joint, and a flexion/extension angle at the left elbow joint, respectively. When he lowered his arm toward the -*z* direction and turned his palm to the *y* direction with the elbow extended, q1, q2, and q3 were all zero, and q4 was π radians.

Patients executed the tasks in an electromagnetically shield room approximately one week after electrode implantation. All patients were seated upright on a chair at a table and were asked to perform the tasks using their left hands. Patient 1 repositioned three blocks around a 25 cm × 25 cm square one by one and in a clockwise fashion (green arrows in Figure 1). He moved his hand to the first block (a rectangular parallelepiped in Figure 1), grasped it, carried it to the vacant corner of the square, and released it. Next, he moved the second block (a cube) to the corner vacated by the rectangular parallelepiped. Finally, he moved the third block (a cylinder) to the corner vacated by the cube. When all objects had been moved to the next corner once, a cycle of hand motion was completed. Patient 1 regularly repeated nine cycles in session 1 and eleven cycles in session 2. Patient 2 also carried the three blocks to vacant corners of the square, but he randomly chose one block among the three to move. Patient 2 performed similar arm movements 19 and 20 times for sessions 1 and 2, respectively. Patient 3 chose one of three blocks and placed it at an arbitrary position on the table. He performed 18, 31, and 24 movements in sessions 1, 2 and 3, respectively. We instructed patients to perform the tasks at their own pace. Each session started just after an audio cue, delivered through a speaker controlled with a MATLAB R2007b (Mathworks, Inc., Natick, MA, USA) script, and continued for 180 seconds. We excluded 20 trials in which patient 2 moved more than 20 cm sagittally because his torso swung forward and backward during the tasks. The abovementioned tasks included several actions, i.e., reaching, grasping, carrying and releasing, which are basic and indispensable actions for daily life.

### ECoG Signals and Motion Recordings

**Figure 2:**
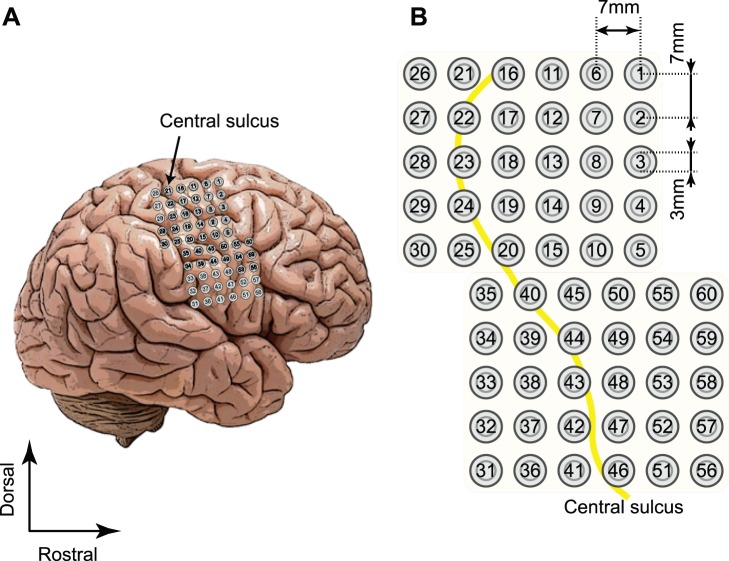
Electrodes placed on the sensorimotor cortex of patient 1. (A) Positions of the electrodes (circles). (B) Two 5 × 6 electrode arrays were placed on the right hemisphere, covering the sensorimotor cortex. Yellow lines depict the right central sulcus.

Patients 1 and 2 were implanted with two 5×6 electrode arrays, and patient 3 was implanted with a 3×5 array. The planar-surface platinum grid electrodes had a diameter of 3 mm and an inter-electrode distance of 7 mm, as shown in Figure 2. The number of electrodes was 60 for patients 1 and 2, and 15 for patient 3. ECoG signals were recorded inside an electromagnetically shielded room with a 128-channel digital EEG system (EEG 2000; Nihon Koden Corporation, Tokyo, Japan) set at a sampling rate of 1000 Hz. All electrodes were referenced to a scalp electrode on the nasion of each patient. Figure 2A shows electrodes placed on the cortex of patient 1.

3D arm motions were recorded at a sampling rate of 100 Hz with an optical motion capture system (Eagle Digital System; Motion Analysis Corporation, Santa Rosa, CA) using reflecting 3D markers shaped in 6 mm-diameter spheroids to identify the left shoulder, left elbow, and left wrist joint positions (Figure 1). The frame lengths of images available for leave-one-out cross-validation (LOO-CV) were as follows: 180 seconds for each session by patient 1, 120 seconds for each session by patient 2, and 90, 180 and 120 seconds for sessions 1, 2, and 3 by patient 3, respectively. Frame lengths differed between patients and sessions since the 3D markers occasionally went out of the field of view or were occluded by the patient’s body. The start of ECoG and motion capture recordings was time-locked to the cue signal.

### ECoG Signal Processing

**Figure 3:**
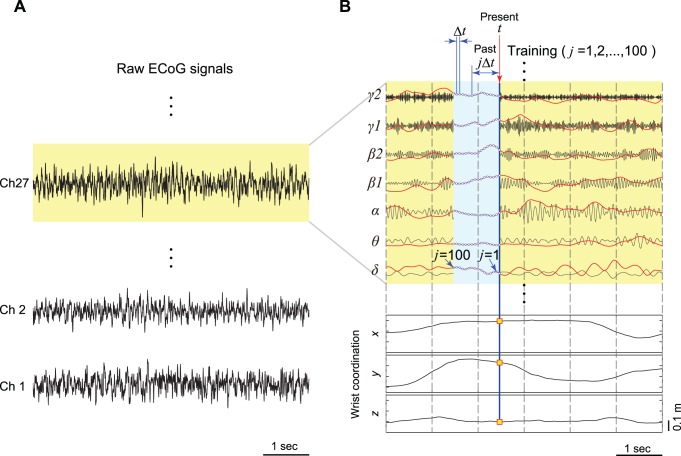
ECoG signal processing and decoding method. (A) Raw ECoG signals from channels 1, 2, and 27 are shown as typical examples. (B) The ECoG signal of channel 27 was divided into seven frequency components (δ,*θ*, …, *γ* 2) with bandpass filters (black lines). These seven filtered signals were digitally rectified, smoothed with a low-pass filter, and down-sampled to 100 Hz. The band-passed ECoG signals were then *z*-score normalized (red lines). The linear relationship between the past 1 s of normalized ECoG (light-blue area; *t* ∼ *t*-*j*Δ*t*, *j* = 1, 2, …, 100, Δ*t* = 0.01 s, i.e., 100 sampling points) and a coordinate *x*, *y*, or *z* at the present *t* (tiny yellow boxes) was determined using sparse linear regression. Once weight coefficients were obtained through training, construction of the decoder was complete.

ECoG signals were pre-processed with our previously proposed method [44]. Firstly, the signal data sampled at 1000 Hz were re-referenced with a common average reference (CAR) and divided into seven frequency bands (δ : ∼4 Hz, *θ* : 4∼8 Hz, *α*: 8∼14 Hz, *β* 1∶14∼20 Hz, *β* 2∶20∼30 Hz, *γ* 1∶30∼50 Hz, and *γ* 2∶50∼90 Hz) using fourth-order bandpass Butterworth filters (Figure 3). Secondly, these band-passed signals were digitally rectified and smoothed with a second-order low-pass filter (cut-off frequency: 2.2 Hz), which changed high oscillations into low frequency features. Thirdly, the signals were down sampled to 100 Hz, i.e., the sampling rate of the motion capture recordings. Finally, the obtained signals *x_i_*(*t*) (*i* = 1, 2, …, *n* 7) at time *t* were normalized to the standard *z*-score *z_i_*(*t*) as follows (red lines in Figure 3B).
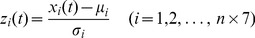
(1)where µ*_i_*, σ*_i_* and *n* denote the mean value of *x_i_*(*t*), the standard deviation of *x_i_*(*t*), and the number of ECoG channels, respectively. These *z*-scores calculated from ECoG signals were utilized as training data to construct a decoder.

### Decoding Method

The value of an angle or a coordinate *Y_p_*(*t*) at a present time *t* was predicted with the following linear equation:
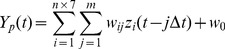
(2)where Δ*t* and *m* denote time-step and the number of consecutive sampling points before the present time *t* used to predict *Y_p_* at *t,* respectively. In this study, we assigned 100 points and 0.01 seconds to *m* and Δ*t*, respectively. *w_0_* and *w_ij_* are, respectively, a bias term and a weight coefficient to the *i*-th filtered ECoG signal *z_i_* at time *t*-*j*Δ*t* (Figure 3B). We applied a Bayesian algorithm called sparse linear regression [44], [46]–[49] to determine values of the weights *w_ij_*.

**Figure 4:**
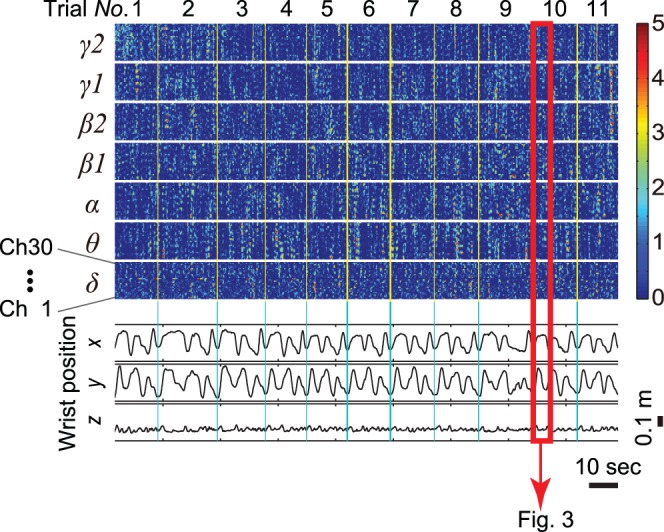
Color-map of the normalized ECoG signals and coordinates at the left wrist joint. Signals were obtained from channels 1∼30 in session 2 of patient 1(channels 31∼60 are not shown). This session includes 11 cycles. We treated each cycle as an independent trial.Start and end points were respectively defined as the instances where tangential velocity of the arm exceeded or fell below 5% of maximum velocity. Unused sampling points are colored yellow (yellow vertical lines). Precise wave forms of *z*-score on channel 27 inside of a red rectangle were already displayed in detail in Figure 3.

Each session was segmented into 9∼31 trials. Figure 4 shows *z*-scores and coordinates *x*, *y* and *z* at the wrist joint in session 2 of patient 1. In this example, the session was divided into 11 trials. We defined the starting point of each trial as the instance when tangential velocity at the elbow joint exceeded 5% of the maximum velocity in the trial. The end point of each trial was decided in a similar manner, i.e., the instance when tangential velocity decreased to less than 5% of maximum. In Figure 4, unused data between the *k*-th ending point and the *k*+1-th starting point are colored over with yellow (yellow vertical lines).

We verified the validity of our method using LOO-CV. Firstly, a decoder was constructed using filtered ECoG signals and actual arm position or actual joint angle in all trials except the *k*-th trial, which was used as test data. The weight coefficients *w_ij_* were obtained from this training. Iterations of the sparse linear regression were terminated just before over-training. Secondly, an arm trajectory *Y_p_* in the *k*-th trial was predicted with the decoder. Pearson’s correlation coefficient (CC) and the normalized root-mean-square error (nRMSE) were obtained by comparing *Y_p_* and *Y_act_* of the *k*-th test trial. Thirdly, the abovementioned training and testing phases were repeatedly executed using different trials for *k* (Figure 4, *k* = 1, 2, …, 11). Finally the CC and nRMSE values were averaged across all trials.

## Results

### Reconstruction of Angles and Positions

**Figure 5:**
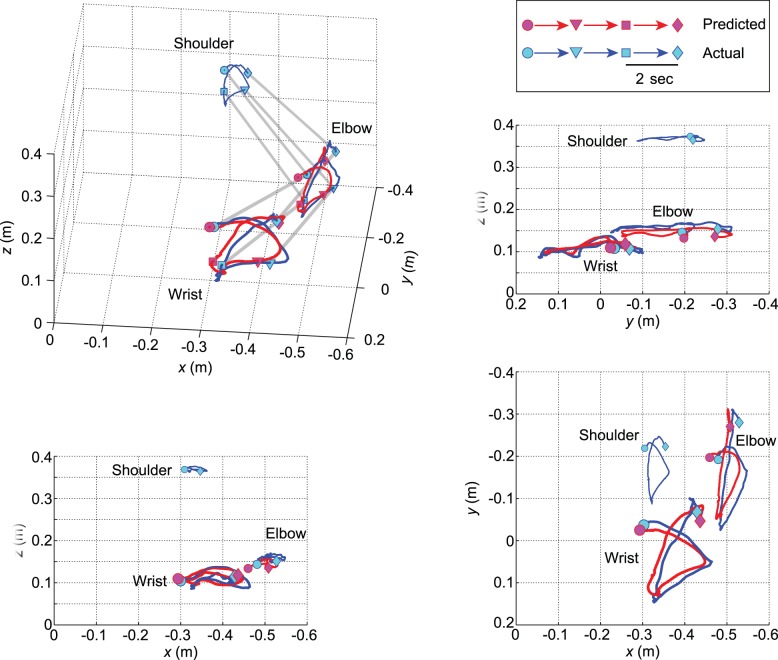
Examples of the predicted (red lines) and actual 3D trajectories (blue lines). A part of the 10th trial (6 s) in session 2 of patient 1 is shown (see Video S1). Markers (circles, triangles, squares, and diamonds) represent 2 s time intervals. Circles and diamonds indicate the earliest and the latest positions, respectively. The red trajectories were computed using predicted data q1∼q4 and patient 1′s actual arm length. The timings (positions of the markers) and trajectory curves of the predicted data were similar to those of the actual data.

**Figure 6:**
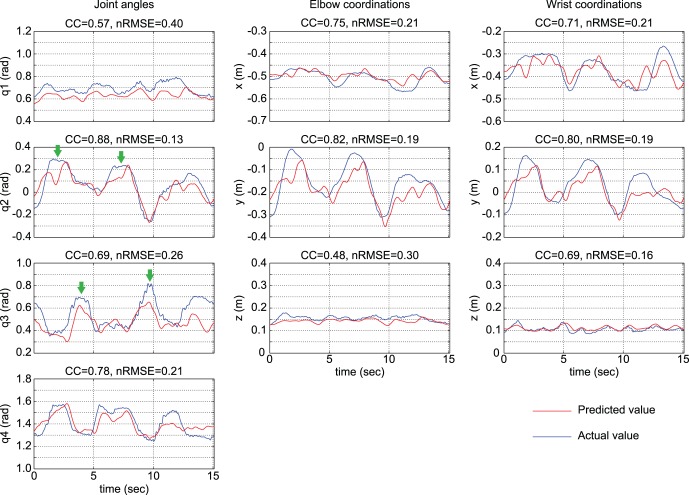
Examples of predicted joint angles and positions in time series. Blue lines are actual recoded joint angles (left column), and actual positions at the left elbow (center column) and left wrist joint (right column) in the 10th trial shown in Figure 3 and Figure 4. The joint angles and coordinates predicted with sparse linear regression are plotted in red. The Pearson’s correlation coefficient (CC) and the normalized root-mean-square error (nRMSE) are shown at the top of each graph.

Movement duration average and standard deviations across 20 trials for patient 1 was 17.17±2.76 s, indicating that his motion in each trial was non-uniform (see Fig. S1). Figure 5 is an example of the comparison between predicted (red lines) and actual 3D trajectories (blue lines) for six seconds in the 10th trial of session 2 by patient 1. The red lines were drawn using inferred joint angles q1∼ q4 and the patient’s arm length. Figure 6 shows predicted joint angles (red lines in the left column) and joint positions (red lines in the center and right columns) in comparison with actual measurements (blue lines) in the 10th trial of session 2 as typical plots by patient 1 (Figure 4). In this trial, it took 15.1 s to move all three blocks to the next open corners of the square. Most blue lines have curvatures with three peaks representing the three block moving tasks. The timings of the peaks differed between q2 and q3 indicated by green arrows. The predicted red lines fit the peaks at various timings, even though the ECoG signals utilized for the prediction were common between q2 and q3. The traces for q1, *z* at the elbow, and *z* at the wrist have narrow variation ranges and many peaks, in contrast to those of the other joint angles/coordinates. The ranges of CC and nRMSE for joint angles (left column in Figure 6) were 0.57∼0.88 and 0.13∼0.40, respectively. The flexion/extension angle q2 at the left shoulder showed the best result. CC and nRMSE for joint coordinates (middle and right columns) were 0.48∼0.82 and 0.16∼0.30, respectively. The *y* coordinate values at the elbow were relatively greater than those of the other coordinates. Both q2 and *y* at elbow showed wider ranges of variation than the others.

**Figure 7:**
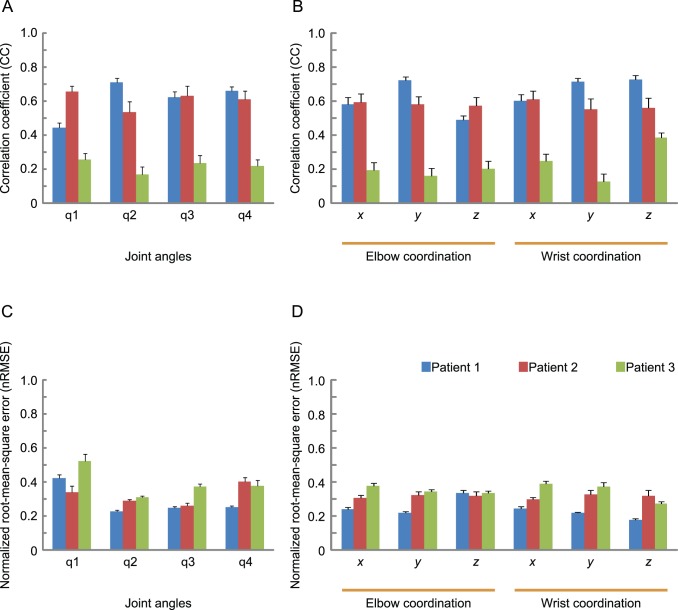
Prediction results for all patients. Averaged correlation coefficients (CC) for joint angle (A) and *x*, *y*, *z* coordination (B), and the normalized root-mean-square error (nRMSE) for joint angles (C) and *x*, *y*, *z* coordination (D) were obtained using LOO-CV on 20, 19 and 73 trials for patients 1, 2, and 3 (blue, red, and green bars), respectively.

Average CC and average nRMSE of the three patients are summarized in Figure 7. The best average CC and nRMSE among joint angles were 0.71±0.026 and 0.23±0.010 (mean ± SEM), respectively, corresponding to angle q2 for patient 1. The best average CC and nRMSE among joint coordinates were 0.73±0.022 and 0.18±0.0071, respectively, corresponding to the *z* coordinate of the left wrist for patient 1.

To judge whether performance of the proposed method differed significantly between patients, a two-way ANOVA with Tukey’s multiple-comparison test was conducted to analyze the effects of two factors (patients and joint angles; patients and joint coordination). The 2-way interaction did not show any significance. Significant differences were observed among the patients (joint angle: *F*_2, 436_ = 82.46, *p*<0.001; coordination: *F*_2, 654_ = 117.56, *p*<0.001), whereas significant differences were not observed among joint angles and joint coordination. The CC values of both patients 1 and 2 were significantly higher than those of patient 3. The nRMSE values for patient 3 were also significantly higher than those of the other patients (joint angle: *F*_2, 436_ = 10.42, *p*<0.05; coordination: *F*_2, 654_ = 41.14, *p*<0.01). This may be interpreted such that the proposed method is more suitable for patients 1 and 2 than for patient 3.

### Frequency Components Contributing to Reconstruction of Arm Trajectory

**Figure 8:**
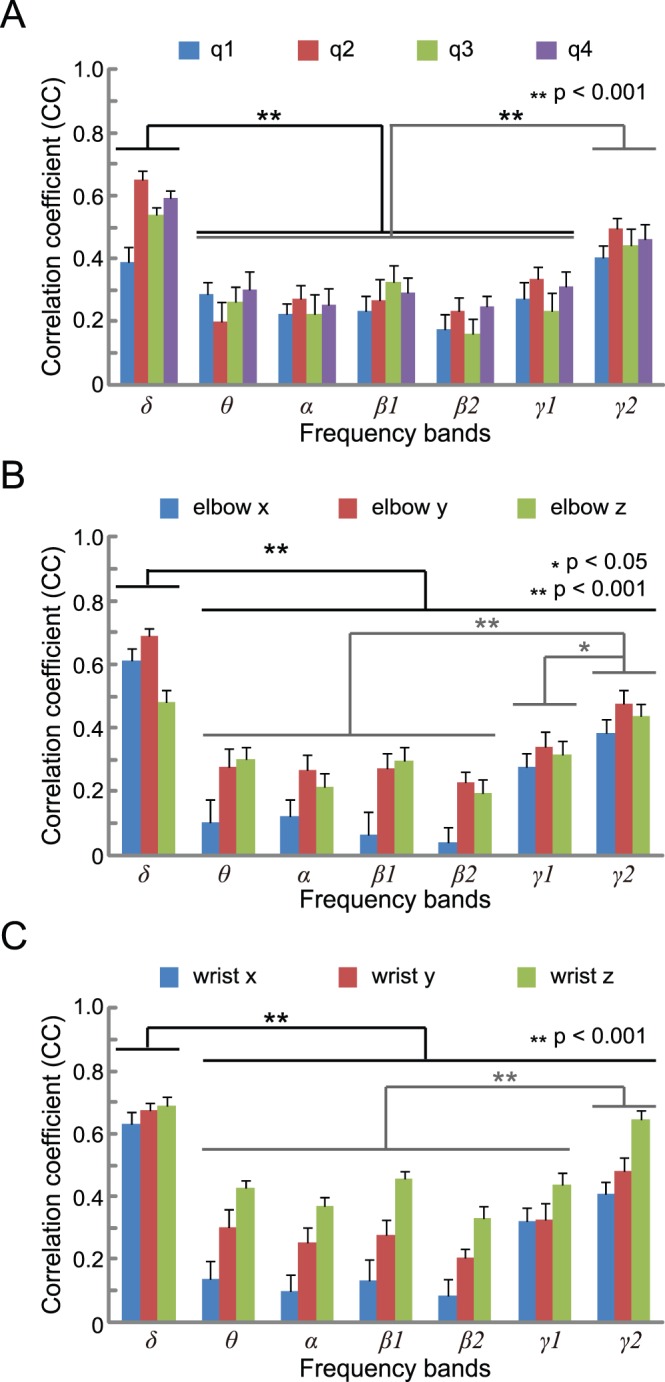
Contribution of each frequency band for trajectory prediction. Each panel (A: joint angles; B: *xyz* coordinates of the elbow; C: *xyz* coordinates of the wrist) shows the results of prediction using each sensorimotor rhythm, one by one. Noteworthy significant differences between CC values of frequency bands are marked with * (p<0.05) and ** (p<0.001). Other significance comparisons are omitted for visualization purposes.

3D hand trajectories were predicted using each sensorimotor rhythm, one by one. The results averaged across 20 trials for patient 1 are shown in Figure 8. A two-way ANOVA was employed to judge two effects (seven sensorimotor frequency bands and four joint angles or six coordinations). Among the 2-way interactions, only elbow coordination showed significance (joint angle: *F*_18, 532_ = 1.07, *p* = 0.38; elbow coordination: *F*_12, 399_ = 1.86, *p* = 0.04; wrist coordination: *F*_12, 399_ = 1.4, *p* = 0.16). Significant differences were observed among the sensorimotor frequency bands (joint angle: *F*_6, 532_ = 27.26, *p*<0.001; elbow coordination: *F*_6, 399_ = 33.67, *p*<0.001; wrist coordination: *F*_6, 399_ = 43.58, *p*<0.001), as shown in figure 8. The CC values of the δ and γ2 bands were significantly higher than those of the other bands.

## Discussion

We predicted 3D arm trajectory in humans based on ECoG signals divided into seven frequency bands using a sparse linear regression method. Although two-dimensional (2D) cursor trajectories on a display have been precisely predicted using ECoG signals obtained from patients in several studies [35], [37]–[38], to the best of our knowledge, inference of 3D trajectory for the human arm using ECoG has not been previously presented.

We inferred both joint angles (q1∼ q4) and joint positions (*x*, *y* and *z*) to reconstruct 3D trajectory and obtained acceptable prediction accuracies in both cases. Our average CC and nRMSE were 0.44∼0.73 and 0.18∼0.42, respectively, excluding patient 3. In the previous studies on 2D cursor trajectories with humans, average CC were approximately 0.22∼0.71 for Schalk et al. (2007) (with the average across positions and velocities for the best participant being 0.62) [35], 0.3∼0.6 for Pistohl et al. (2008) [37], and 0.52∼0.87 for Gunduz et al. (2009) [38]. Kubanek et al. (2009), who predicted individual finger flexions, showed an average CC of 0.23 (little finger) ∼ 0.75 (thumb) (CC averaged across all fingers and participants was 0.52) [41]. Our results were not inferior to the aforementioned studies, especially considering the higher dimensionality of trajectory data.

The prediction accuracy for patient 3 was significantly worse than that of the other patients. His average CC and nRMSE were 0.13∼0.38 and 0.28∼0.52, respectively. We suggest the following as possible causes for this result: (1) ECoG signal quality; There were obvious disturbances or noise in his ECoG signals which could be discerned through visual inspection. The baselines of his ECoG signals also randomly and widely fluctuated. (2) Electrode number; Patient 3 had only 15 electrodes placed around his central sulcus, whereas the other patients had 60 electrodes. (3) Pathology; Patient 3 had epilepsy while the others did not. (4) Task properties; He was allowed to place the blocks at arbitrary places on the table. He decided their positions impromptu, in contrast to the other participants who placed their blocks at fixed positions. We suggest that much more training data are necessary for the prediction of motions involving various postures such as those in the data of patient 3.

Joint angle q1 could not be predicted precisely, in contrast to q2∼q4 (Figure 7A and 6C). The range of abduction/adduction for q1 was the narrowest among all angles, as shown in the left column of Figure 6. We presume that it was difficult to extract the faint component correlating with this small fluctuation from ECoG as a summation of various signals.

The high frequency band γ2 (50∼90 Hz) had relatively high CC values (Figure 8). Several papers also reported that high frequency bands of ECoG were important for prediction, such as 40∼80 Hz for cursor trajectory prediction in humans [37], 80∼150 Hz for the classification of human hand movements [31], 40∼90 Hz for 3D hand trajectory prediction in monkeys [43], and 50∼90 Hz for EMG prediction in monkeys [44]. The low frequency band δ (∼4 Hz) had the highest values among the seven bands in this study. This was also supported by previous works [32], [37] which reported that the low frequency band ECoG (2∼6 Hz; with band-pass filter) and low frequency component (LFC) (<5 Hz; with Savitzky-Golay smoothing filter) were important for classifying different grasp types [32].

We verified that 3D arm trajectories in patients of different pathology could be predicted with our proposed method using a sparse linear regression. We foresee this method contributing to further studies and further improvements in neuroprostheses and neurorehabilitation.

## Supporting Information

Figure S1 Actual position at the wrist joint for patient 1. Coordinates *x*, *y*, and *z* of all 20 trials are shown. Motion of patient 1 was non-uniform, with duration and timing differing between trials.
(EPS)

Video S1 Examples of the predicted arm positions of patient 1. Blue and red lines are actual and predicted arm positions in the 10th trial of session 2, respectively.
(MOV)
